# Exploring Couple‐Based Coping Strategies With Infertility: A Qualitative Study

**DOI:** 10.1002/hsr2.72069

**Published:** 2026-05-03

**Authors:** Marzie Reisi, Ashraf Kazemi, Nafisehsadat Nekuei

**Affiliations:** ^1^ Midwifery Department Shahrekord University of Medical Sciences Shahrekord Iran; ^2^ Reproductive Health Department, Nursing and Midwifery Care Research Center Isfahan University of Medical Sciences Isfahan Iran; ^3^ Reproductive Health Department Isfahan University of Medical Sciences Isfahan Iran

**Keywords:** coping strategies, couple‐based, infertility, qualitative study

## Abstract

**Background and Aims:**

Infertility is a medical condition with associated psychosocial problems on couple's life that requires employing coping strategies using a couple‐based approach. Therefore, the aim of the present study was to explore coping strategies using a couple‐based approach.

**Method:**

This study used a qualitative content analysis approach and semi‐structured interviews with 36 Iranian individuals, selected through the purposeful method and considering maximum diversity in terms of age and cause, type, and duration of infertility, 10 of which couples were conducted in pairs interview. Content analysis was performed using the Granheim and Ludman method.

**Results:**

Three main categories were extracted from data analysis: “coexistence with infertility,” “emotional relief,” and “consolidating couples' relationships.” The coexistence with the infertility category was deduced from consolidating couples’ relationships, participation in accepting the responsibility of fertility, and reorganizing married life themes; the emotional relief category was extracted from the spiritual intimacy, the value of married life, and scenario planning themes, and consolidating couples’ relationships category was obtained from the dynamic interaction and emotional intimacy themes.

**Conclusion:**

The study results showed that coping strategies could be investigated in three general categories: coexistence with infertility, emotional relief, and consolidating couples' relationships. Therefore, it is recommended to consider these coping strategies in designing and providing counseling services to infertile couples.

**Strengths and Limitations of This Study:**

The results of the present study expanded our understanding of adaptive coping strategies, recommending these strategies in counseling programs requires a study with a quantitative approach. But the findings are limited in generalizability to Iranian infertile couples actively seeking fertility treatment.

## 1 Introduction

Reproduction is a significant goal for many couples worldwide, and infertility can be considered a crisis. Approximately 10 to 15 percent of couples of reproductive ages face this disorder [1] and suffer from its psychosocial consequences. Although infertility is a non‐fatal medical disorder, it has become a social problem due to its association with gender identity, gender roles, and social acceptance. Therefore, its diagnosis and treatment using assisted reproductive techniques (ART) put a great deal of stress on couples and expose them to severe tension [2].

In some societies, infertility is associated with stigma [3, 4], isolation [5], increased likelihood of divorce and domestic violence [4, 6]. It can also lead to depression, stress, anxiety [7, 8], decreased self‐confidence [9], psychological adjustment [7], and a sense of inefficiency [10]. Moreover, social pressures caused by the social taboo of infertility can cause a psychological imbalance between couples and, in some cases, weaken their relationship [11, 12]. In addition, the reduction of sexual satisfaction [9] and marital quality and even the emergence of sexual disorders [13] in infertile couples expose their lives to further challenges.

Due to the importance of having children in the sociocultural context of traditional societies, the infertility in these societies includes deeper dimensions and disrupts dealing with it. Domar et al. believe that due to the wide range of psychosocial problems caused by infertility, it is necessary to consider this disorder as severe as other critical physical disorders and a source of chronic stress and apply effective coping strategies to modify its psychological pressures [14].

Coping strategies are a set of cognitive and behavioral efforts used to interpret, analyze, and modify a stressful situation. Lazarus defines coping as an individual effort to overcome harmful, threatening, or challenging conditions [15].

Individuals' strategy to deal with a crisis is influenced by the sociocultural values and norms of the society in which they live. Moreover, their coping is affected by the crisis they are exposed to. From a medical point of view, infertility is a manageable and treatable disorder. However, its social problems, especially in traditional societies, are beyond the control of infertile couples and can lead them to social isolation and frustration.

In dealing with an infertility crisis, each couple may employ different strategies. Nevertheless, infertility is a couple‐based issue, and couples' close and comprehensive interaction facing its psychosocial problems involves both partners and, therefore, causes each partner's coping strategies to affect those of the other. Consequently, couple‐based strategies will be more effective in dealing with infertility and the stress caused by it.

Identifying coping strategies with an adaptive couple‐based approach to modify the infertility crisis in order to develop counseling programs for infertile couples requires identifying the couples' strategies toward each other and in their sociocultural context. Therefore, the aim of the present study was to identify infertile couples' couple‐based coping strategies through a qualitative study.

## 2 Methods and Analysis

This study was conducted using a qualitative content analysis approach with the approval of the ethics committee of Isfahan University of Medical Sciences in Isfahan, Iran, between January and July 2023. The participants in the study included 36 infertile Iranian men and women (an equal number of each) referred to the Isfahan Fertility Center to initiate treatment. Of 36 individuals, 10 were couples and were conducted in pairs face to face interview. The inclusion criteria for the participants included having no children and no severe mental illnesses, such as schizophrenia and psychosis, based on the documents in the file.

Purposeful sampling was employed to ensure maximum diversity in age, educational background, and the duration and etiology of infertility. From among individuals attending infertility centers for outpatient infertility treatment services, eligible participants were identified and invited to take part in the study. Participants were identified and invited to the study after being admitted to the center. After selecting the participants, a reproductive health specialist, one of the female researchers (PhD candidate), who did not work in infertility centers, explained the study objectives and participation. The interviewer was trained in qualitative interviewing techniques to facilitate rapport and ensure cultural sensitivity. Participants were ensured of voluntary participation that would not affect their medical services. It was also explained that their information would be kept confidential, and only the researchers would access the interview transcriptions. The participants had the right to withdraw from the study at any stage. Informed consent was obtained from all the invited individuals.

Data were collected through interviews in a private room at the infertility center. Efforts were made to establish a positive rapport with participants prior to the commencement of the interview in order to foster trust. Based on the participants' willingness, the interview was conducted with or without the spouse's presence. Guiding questions were developed using unstructured in‐depth interviews with 3 couples, and the interviews were continued in a semi‐structured manner using the guiding questions, such as “Describe your experiences of infertility,” “Explain your and your partner's shared feelings regarding infertility,” and “What strategy did you use to prevent this feeling?”

During the interview, an attempt was made to obtain a deeper understanding of the experiences by asking questions such as “Explain more about this feeling.” Moreover, during the interview, emotional reactions and non‐verbal expressions were recorded manually to help gain a better understanding of the narratives during the interview analysis. The main interviewer was one of the female researchers. There was a possibility for the interview to be conducted by the other gender upon the participant's request. The interviews were voice‐recorded using a tape recorder. Before starting the audio recording, permission was obtained from the interviewee, and in case of unwillingness, the interviews were recorded manually.

Based on each participant's conditions, the interview lasted roughly 60 to 90 min. Afterward, the interviewer researcher listened to the interviews several times to gain an overall understanding of the interviews. Then, the interviews were transcribed verbatim. The participant's emotional reactions and recorded non‐verbal expressions were placed next to the related text. Data analysis was performed for each interview simultaneously with sampling. The interviews continued until the data were saturated, and no inferential codes and new subcategories were extracted.

### 2.1 Data Analysis

To analyze the data, qualitative content analysis was performed manually using an inductive approach and the Grandheim and Ludman method [16]. Qualitative content analysis is a suitable method for data analysis in research that aims to describe important topics for a particular group. Since individuals' experiences are interpreted in the context in which they live, this analysis method was selected. The interview was considered as the unit of analysis. The stages of data analysis included determining the content, unit of analysis, and meaning units, compressing and summarizing, generation and classification of codes, and formation of sub‐themes, and main themes.

After the interview text was transcribed, it was read several times, and meaning units and primary codes were extracted. In the next step, inferential codes were formed, and similar codes were merged. Similar inferential codes were placed in sub‐themes, and subsequently, and main themes were inferred.

In order to ensure the trustworthiness of the data, four criteria of credibility, dependability, transferability, and confirmability were evaluated. The member check method was used to increase the validity of the findings. To this end, the compatibility of the findings with participants' experiences was evaluated by calling 4 individuals. Moreover, the results and analysis of the findings were provided to three experts in the field of clinical psychology and one individual in the field of reproductive health, who were not a member of the research team, and their complementary and critical opinions were considered. In order to comply with the dependability, all study stages were recorded, and a part of the interview transcriptions was provided in the results report. A purposeful sampling method with maximum diversity was used to meet the transferability. Moreover, to increase confirmability, all study stages were recorded, and documents and reports were maintained.

## 3 Results

Of the 40 individuals invited to participate in the study, 36 eligible participants accepted the invitation. A total of 31 interviews were conducted, involving 18 women and 18 men, 10 of whom were couples. Women's and men's ages ranged between 26 and 40 and 27–44, respectively. The period of infertility was between 2 and 22 years (Table 1).

**Table 1:** Individual characteristics of the participants.

Participant	Age	Gender	Educational level	Employment status	Ethology of infertility	Duration of Infertility (years)
1	36	Woman	Bachelor degree	Employed	Female	5
2	38	Woman	MSc	Employed	Female	12
3	40	Woman	Diploma	Employed	Male	15
4	33	Woman	PhD	Employed	Male	4
5	36	Man	Bachelor degree	Employed	Female	10
6	31	Woman	Diploma	Employed	Female	5
7	33	Man	Bachelor degree	Unemployed	Female	4
8	32	Woman	Bachelor degree	Employed	Male	2
9	41	Man	Bachelor degree	Unemployed	Unexplained	13
10	27	Man	Diploma	Employed	Male	4
11	33	Woman	High school	Employed	Male	12
12	36	Woman	Diploma	Unemployed	Unexplained	3
13	30	Man	Bachelor degree	Unemployed	Female	4
14	39	Woman	Diploma	Employed	Female	2
15	34	Woman	Bachelor degree	Unemployed	Female	5
16	38	Man	MSc	Employed	Unexplained	14
17	34	Man	Diploma	Employed	Male	6
18	34	Woman	Diploma	Employed	Female	10
19	31	Woman	MSc	Unemployed	Unexplained	3
20	39	Man	Diploma	Unemployed	Female	5
21	27	Man	Diploma	Employed	Male	3
22	34	Man	Diploma	Unemployed	Male	4
23	44	Man	Bachelor degree	Employed	Unexplained	22
24	38	Man	High school	Employed	Female	19
25	38	Woman	High school	Employed	Unexplained	9
26	33	Man	PhD	Employed	Female	2
Couple 1						
27	38	Woman	High school	Unemployed	Female	15
28	39	Man	High school	Employed	Female	15
Couple 2						
29	32	Woman	Bachelor degree	Unemployed	Male	4
30	35	Man	MSc	Employed	Male	4
Couple 3						
31	36	Woman	Bachelor degree	Employed	Female	10
32	41	Man	Bachelor degree	Employed	Female	10
Couple 4						
33	32	Woman	Bachelor degree	Employed	Male	2
34	36	Man	Bachelor degree	Employed	Male	2
Couple 5						
35	29	Woman	Diploma	Unemployed	Male	5
36	37	Man	Diploma	Employed	Male	5

Qualitative data analysis led to the extraction of 513 inferential codes, 16 sub‐subcategories, 8 subcategories, and 3 main categories. The main categories included coexistence with infertility, emotional relief, and consolidating couples’ relationships (Table 2).

**Table 2:** Main themes and sub themes.

Main themes	Sub themes	Meaning codes
Coexistence with infertility	A realistic view of infertility	Infertility as an undeniable fact
		Coming to terms with the prolonged treatment journey
		Acknowledging the societal dimensions of infertility
		Accepting the unpredictability of treatment results
	Participation in taking responsibility for fertility	Acceptance of infertility as a couple's problem
		Understanding the need for couple participation in infertility treatment
		Couples' lack of focus on the cause of infertility
		Viewing infertility as one of the marital life challenges
	Reorganizing married life	Downplaying the importance of infertility
		Rethinking marital lifestyle
		Regaining balance in life
		Expanding social relationships
Emotional relief	Spiritual intimacy	Confirmation of each other's spiritual appeal
		Joint participation in performing spiritual rituals
		Joint participation in carrying out charitable works
	The value of married life	Paying attention to the positive aspects of living together
		The value of living together
		Focusing on mutual love and intimacy
		Procreation as a secondary goal of living together
	Scenario planning	Expressing of shared wishes
		Expressing ways to make dreams come true
		Sharing wishes for future children
Consolidating couple relations	Dynamic interaction	Honest Interaction
		Joint Effort to Maintain Hope
		Couple Empathy
	Emotional intimacy	Joint effort to control negative thoughts
		Scenario‐making and visualizing a better future
		Maintaining peace and contentment

### 3.1 Coexistence With Infertility

This main category was derived from the three subcategories: a realistic view of infertility, participation in taking responsibility for fertility, and reorganizing married life.

#### 3.1.1 A Realistic View of Infertility

This subcategory was inferred from the themes that referred to the couple's effort to reach a mutual understanding of infertility and included considering infertility as a disorder, not a defect, and avoiding exaggeration of this disorder. In this regard, participant number 8 stated: “We were completely shocked when we found out that we had a problem having a child. It was very hard. The life we loved so much was going to fall apart. We felt we weren't a perfect couple to have a good life. We avoided finding out which one of us had a problem. We could only deal with this problem when we started to believe that others could have children without help, but we need to be treated with doctors' help, like many other patients.”

Avoiding exaggerating infertility was also extracted from the statements that showed the couples, in their interaction, made an effort to underestimate the importance of infertility; consequently, the problem could not overshadow their married life. In this regard, the participant number 12 said: *“My husband and I loved and still love children very much. It is important for us to be able to have our own child, but not so much that our married life is ruined because of not having one.”*

#### 3.1.2 Participation in Taking Responsibility for Fertility

The research data showed that one factor helping couples' coexistence with infertility was participation in taking responsibility for fertility. This theme was extracted from the statements showing that, regardless of the cause of infertility, if both men and women considered fertility as a result of married life and did not attribute it to each other, they were more compatible with the conditions. In this regard, participant number 4 said: *“My husband got really upset when we found out that the reason (infertility) was his low sperm count. Before the cause of infertility was diagnosed, he was upset too, but not as much as when the doctor explained the test results (spermogram) to him; he was nervous and said: “The problem is me, do you understand?” I kept telling him: “It doesn't matter at all! What is important is our attempt to have children, me and you; to have a child, you and I (emphasis) need to struggle.” When he heard these, he calmed down.”*

#### 3.1.3 Reorganizing Married Life

Another subcategory of coexistence with infertility was reorganizing married life, formed from the subcategories revising lifestyle, revising married life goals, developing social relationships with peer groups, and avoiding social pressures.

Revising lifestyle was extracted from the statements indicating that infertile couples struggled to strengthen other dimensions of married life, spend more leisure time together, and plan for joint side activities to deal with infertility. In this regard, participant number 14 said: *“Well, both of us are very fond of exercising. My husband told me that since we had free time, it was better to enjoy ourselves and do the activities we wouldn't have time for after having children. He suggested, and I accepted, and we started going mountain climbing, which seems to have become a habit.”*

Revising married life goals was likewise deduced from the statements indicating that in order to coexist with infertility, couples shifted the goal of raising a child to other goals in their married life and designed new joint goals. Regarding that, participant number 15 said: *“When two people get married, others think that the next stage of their married life is to have a child, which bothered me and my husband. We became nervous when we thought we couldn't succeed in the life we started together. But finally, we concluded that we had many plans for the future.”*

Furthermore, developing social relationships with peer groups was extracted from the experiences showing that couples made an effort to gain a more realistic social understanding of infertility and not consider infertility exclusively theirs by developing social relations with other infertile couples. Besides, they appreciated the benefit of their informational support to solve problems related to infertility. In this regard, participant number 2 said: *“In the beginning, I felt terrible because the problem was with me.” But since I talked to people who came for treatment like us in the (fertility and infertility) center, my mood improved a lot. I saw so many couples having the same problems as us. We felt close to them.”*

Avoiding social pressures was another subcategory extracted from the statements showing that in order to maintain their mental health, some couples avoided joining groups that put the psychological pressure of being infertile on them. These social pressures were caused by judgment, being stigmatized as an unhappy couple, and sometimes the compassion felt by others. Regarding that, participant number 11 said: *“When we attended family parties, especially when my husband and I were together, I could notice others' strange looks. I didn't dare to hug a child. I thought that others believed how enthusiastic I was to hug my child. Actually, I wanted to hug my child, but others had no right to think so. These looks bothered me and my husband. Even when they prayed for us to have children, we were annoyed. So, we decided not to attend family gatherings that teased us because of having no children”*.

### 3.2 Emotional Relief

This main category was derived from the subcategories spiritual intimacy, value of married life, and scenario planning.

#### 3.2.1 Spiritual Intimacy

The subcategory of spiritual intimacy was derived from the two themes: couples' companionship in performing spiritual rituals and joint benevolent actions. Some couples planned to perform spiritual rituals jointly to moderate the infertility crisis. Some believed that jointly performing those actions increased their intimacy and provided them emotional relief. Moreover, the spouse's company for benevolent activities caused them to feel that their spouse likewise cared about the partner's spiritual health and accompanied him/her. In this regard, participant number 4 said: *“Having a child has become a concern for me and my husband. So we both have to pray together. We play prayer audio tape and pray together for a while. Having the same desire and asking it from God gives us peace. It is very valuable to me when he accompanies me while praying. This way, he actually approves me.”*

Some couples participated in charity work jointly to achieve spiritual intimacy. Among the benevolent activities, taking care of children caused more pleasant feelings in couples than other activities. In this regard, participant number 8 said: *“We used to go together to the welfare administrations that cared for the orphans. When we took care of them, we were all filled with joy. I gave them my motherly love.”*

#### 3.2.2 The Value of Married Life

This subcategory was extracted from the sub‐subcategories of authenticity of married life versus parenthood and romantic life. Some participants stated that their married life was so important that the problem of infertility could not destroy their peace of life. In this regard, couples who were a priority for their spouses reported higher peace. Regarding this, participant number 20 said: *“Well, it is true that having children is good, but for me, my wife's peace is more important. We are trying to have children, but not at the cost of my wife's discomfort. I won't let not having children put a distance between us. I always tell my wife that our life and love have a higher value. When my wife sees that my love for her hasn't diminished over time, she copes with infertility more easily. Her peace brings me peace.”* The romantic life theme was deduced from the participants' statements, implying that the couple's attention to the interest and love they had for each other and the effort to express romantic feelings made them better tolerate the lack of children. In this regard, participant number 21 said: *“We started our life with love. At first, the infertility problem occupied our minds and made us ignore each other. Of course, part of it (decreased expression of interest) was because several years had passed since our marriage. But our problem (infertility) made us both think that not having a child caused us to express less love to each other. So we decided to remind each other daily how much we love each other and how important it is for us to be together.”*

#### 3.2.3 Scenario Planning

This subcategory was formed from the sub‐subcategories of playing the parental role and sharing dreams about the future child. It was extracted from the statements showing that infertile couples planned scenarios of child care and having a child to relieve themselves emotionally. They distanced themselves from thinking about infertility by sharing their scenario and desires for their future child. Regarding this, one of the participants said: *“When thinking about infertility bothers me, I fantasize. I visualize an imaginary child for myself, and I talk to it; I complain to his/her father, and my husband defends him/her (smile). It calms me down.”*

Participant number 18 stated: *“No one knows about the future. The future is what we think about today. Because of that, I have dreams for the future child I talk about with my husband. He also talks about his dreams. We once argued about what job we want our child to get (laughter).”*

## 4 Consolidating Couple Relations

This category was extracted from the subcategories dynamic interaction and emotional intimacy.

### 4.1 Dynamic Interaction

This subcategory was formed from the sub‐subcategories of sharing feelings, positive emotional reactions, and understanding each other's needs, and from the statements showing that an interaction based on mutual understanding could moderate the psychological pressures caused by infertility. In this regard, participant number 6, who was upset with the way her husband interacts with her when she is distressed, said: *“I need to talk to my husband so my feelings are not suppressed. I feel alone when my husband criticizes my feelings and attributes my emotional problems to me (pause). We have no common subject to talk about. He doesn't see me at all (crying). I just want him to understand me, to tell me he's there and understands what I'm saying.”*

Moreover, participant number 17 said: *“Because the infertility problem had affected us a lot, we were depressed. We visited a consultant. He gave us good advice. He asked us to restore our relationship, understand each other's needs and desires, be honest with each other, and not show any hasty reaction to each other's feelings and what we tell each other. He was right. When my wife was upset or angry, I would get angry, too, and we would argue. The consultant said that if her feelings were hurt, I should have looked for the cause (pause). One of his sentences was interesting to me. He said to me to be a wise and empathetic listener to my wife.”*

### 4.2 Emotional Intimacy

Emotional intimacy was one of the subcategories of the consolidating couple's relationships, extracted from the themes of developing romantic behaviors and spending more time together. These sub‐subcategories were deduced from the statements indicating that the couples tried to satisfy each other's emotional needs through romantic behaviors, such as kissing, hugging, and expressing love. Moreover, spending time with each other was one of the participants' efforts to increase mutual intimacy. In this regard, participant number 22 said: *“The infertility problem bothered both of us. We were both under pressure and needed to reduce the pressure. We thought about why we didn't spend the time we had to spend on children on each other. Maybe we would have less time to make love if we had children. Therefore, this part of our married life became more important for us and increased our intimacy.”*

## 5 Discussion

The present study was conducted to identify infertile couples' coping strategies. It indicated that couples dealt with the infertility crisis by coexisting with infertility, emotional relief, and consolidating their relationship.

A realistic view of infertility, participation in taking responsibility for fertility, and reorganizing married life formed the coexisting infertility category, indicating that the couple's effort to reach a common understanding of infertility as an undeniable fact assisted them in continuing their married life less stressfully despite being infertile. The results showed that by accepting differences from others, couples attempted not to deny their capability to have children and moderate the negative feelings following infertility by realistically considering infertility and admitting the difference. In this regard, a study has shown that the normalization and efforts of infertile couples to enjoy a customary yet different life are associated with a higher quality of life and psychological adjustment [17].

Moreover, couples' logical understanding of the problem of infertility as a shared problem and participation in taking responsibility for fertility helped couples participate in solving this problem and reduce the feeling of guilt in each other. The abdication of fertility responsibility by one of the couples may challenge and reduce the couple's adaptation to infertility by inducing insecure attachment.

A study on factors related to stress in infertile couples has shown that the level of stress was higher in the partner who was the cause of infertility [18]. This stress could result from the feeling of shame following the inability to conceive [19, 20]. However, a phenomenological study on the experiences of couples undergoing infertility treatment has shown that couples' involvement in joint hardship is one of the opportunities expressed by couples to strengthen their marital bond [21]. Confirming these reports, the present study showed that taking responsibility for fertility in couples is an adaptive strategy for better adaptation to infertility.

Another finding of the study showed that an attempt to reduce the significance of having a child rather than other goals of married life was one of the strategies used by infertile couples to deal with infertility and improve the quality of their married life despite infertility. In societies where having children is an important goal of a marital relationship, the importance of fertility can challenge the infertile couple's sense of competence to play gender roles and endanger their mental health. In this regard, a study has shown that those who considered having children a central goal and committed to achieving this goal experienced more stress [22]. It has also been reported that a couple's lack of understanding of life without children was one of the risk factors for social concerns in couples with infertility problems [23].

The results of the present study confirm the results of the study by Neter et al., which showed that individuals' high ability to ignore the goal of having children had a protective effect against the distress caused by infertility and their inability to formulate new goals was associated with more suffering [24].

Another finding that formed the concept of coexistence with infertility was the reorganizing married life theme, which showed that infertile couples were making an attempt to adapt to infertility by revising their lifestyle, goals of marital life, and social relationships. This finding shows that infertile couples need to change their lives according to the conditions in order to moderate the infertility crisis. One of these changes adopted by couples in their social communication was to avoid family gatherings to overcome the social pressures of infertility and to strengthen their relationships predominantly with infertile couples.

Although social relationships with the family can reduce the psychological burden of infertility by strengthening the understanding of social support, reports show that in some traditional societies, infertile women are criticized by their relatives, which is associated with increased marital tensions [25, 26].

Social isolation and psychological problems following the disclosure of infertility have already been reported [26]. Infertile couples need to strengthen their relationship with the peer group, which confirms the study results indicating that group‐based psychological interventions are an efficient way to reduce infertility stress [27]. Confirming these reports, the results of the present study showed that the psychosocial burden of infertility, such as stigma and social pressure impelled infertile couples to avoid the communities and relationships that exposed them to these problems and to strengthen their relationships with groups that have shared concerns [28].

The other category resulting from the data analysis was emotional relief, formed from the subcategories of spiritual intimacy, value of married life, and scenario planning. The spiritual intimacy theme was deduced from statements showing that couples coped with infertility by participating in religious activities, charity work, and mutual respect for each other's religious beliefs and tried to give meaning to their married life. They also attempted to alleviate the negative feelings caused by infertility by strengthening the spiritual dimension of life as a common goal.

Hess et al. reported that in Mali, infertile women referred to religious practices due to their social stigma and exclusion [22]. The research showed that once this approach was taken jointly, it would probably be associated with more positive feelings in the couple. Some documents show that spiritual intimacy is related to marital satisfaction [29] and marital intimacy. Mahoney et al. reported that sharing spiritual views by couples was associated with reduced verbal hostility [30].

The results of this and other studies show that spiritual affairs as an individual strategy have protective effects on infertile women's and men's mental health [31]. Infertile couples' mutual respect for beliefs creates spiritual intimacy by creating emotional relief. Furthermore, the couples' participation in benevolent affairs provides an opportunity for more intimacy between couples and relieves them of stress.

In addition, the results of the present study showed that one of the infertile couple's strategies for emotional relief was valuing married life. This study finding showed that some couples struggled to consider their married life independent of fertility and strengthened this understanding to be able to control the psychological emotions of infertility.

Furthermore, by strengthening their romantic relationships, some couples strived to replace the love of having children with positive emotional feelings. Other studies have also shown that romantic relationships between couples [32, 33] and the quality of marital relationships in infertile couples [34] have been associated with the reduction of psychological complications. This finding shows that an effort to improve communication skills and marital relations is probably an effective strategy to improve infertile couples' mental health.

In addition to attempting to increase spiritual intimacy and understanding the value of married life, joint scenario planning in infertile couples was one of the strategies to achieve emotional relief. This subcategory was extracted from experiences showing that couples consoled themselves by expressing their dreams and fantasizing and planning for the arrival of a child. These results show that some infertile couples visualize the parental role by fantasizing and creating scenarios, and the participation and companionship of a partner confirm another partner's qualification to play the parental role. These couples strengthen their positive thoughts and obtain peace by applying this strategy.

In this regard, Fata and Tokat similarly reported that fantasizing and creating positive thoughts in women undergoing fertility treatment was associated with decreased blood cortisol [35]. Although scenario‐creation and imagination, as positive thoughts through the interaction between mind and body, can be associated with stress reduction [36] and provide peace for the couple, it should be noted that drowning in fantasy may deprive the couple of the ability to successfully cope with the treatment and may distance them from reality.

Another study finding was the consolidating couple relations category, formed from the subcategories of dynamic interaction and emotional intimacy. The dynamic interaction subcategory was a concept derived from couples' experiences of empathizing with each other and understanding and sharing feelings. Previous research has shown that sexual partners' mutual understanding of their partner's social concerns is one of the protective factors against their depression and anxiety [23]. Molgora et al. had shown that dyadic positive coping, including emotion‐based coping, helps improve infertile couples’ adaptation [37].

It has also been reported that in infertile couples, the spouse's perceived marital coping reduces the negative impact of infertility on their emotional adaptation [38] and has been associated with a higher quality of life and mental health [39]. Dynamic interaction between couples can strengthen the perception of psychosocial support in couples and reduce the stress caused by infertility [40]. The results of the present and these studies confirm that an attempt to have a positive interactive relationship between couples can improve adaptation to infertility by consolidating couples' relationships.

Emotional intimacy was another concept associated with consolidating couples' relationships, which was deduced from statements indicating that couples reduce infertility stress by attempting to have a romantic married life and strengthening emotional intimacy [41]. This finding confirms the results of Peloquin's study, which showed that insecure attachment between infertile couples was associated with less marital satisfaction and marital coping was an inadequate mediator of this relationship [42].

Although the results of the present study expanded our understanding of adaptive coping strategies, recommending these strategies in counseling programs requires a study with a quantitative approach. Moreover, these results are only generalizable to the community of infertile couples since this study was conducted on couples referred to undergo fertility treatments. Therefore, it cannot be generalized to couples undergoing treatment who are not involved in the treatment process. Furthermore, this study was conducted in Iranian society; consequently, it is recommended to conduct further research in other social and cultural contexts.

In conclusion the present study showed that three types of coping strategies, including coexistence with infertility, emotional relief, and couple‐based interaction, were among the couple‐based strategies that Iranian infertile couples used to deal with infertility and interact with each other.

These results suggest that, in addition to strengthening a logical insight into infertility, infertile couples might use the spiritual dimension of married life and visualization of the future as coping strategies to interact with each other and manage the infertility crisis. The results of this study can be used in compiling coping strategies with a couple‐based approach for infertile couples and in planning counseling programs for infertile couples.

## Author Contributions

MR was involved in designing the protocol. She was involved in designing the questionnaire, interpretation of the data, writing, revising and editing of the article. AK was involved in the project development and designing the protocol. She was involved in designing the questionnaire, data collection supervising, acquisition of analysis, interpretation of the study results and article writing and revising. She is responsible for all aspects of the Ethics Committee of the Isfahan University of Medical Science. NN was involved in designing the protocol. She was involved in designing the questionnaire, interpretation of the data and editing of the article. MR, AK and NN wrote the main manuscript text. All authors have read and approved the final version of the article. AK had full access to all of the data in this study and takes complete responsibility for the integrity of the data and the accuracy of the data analysis.

## Ethics Statement

Ethical approval for this study has been obtained by the ethics committee affiliated with Isfahan University of Medical Sciences, Isfahan, Iran. (IR.MUI.NUREMA.REC.1400.019). All the procedures to the participants were in accordance with the ethical standards of the Isfahan University of Medical Sciences.

## Conflicts of Interest

The authors declare that they have no conflict of interest.

## Transparency Statement

The lead author Ashraf Kazemi affirms that this article is an honest, accurate, and transparent account of the study being reported; that no important aspects of the study have been omitted; and that any discrepancies from the study as planned (and, if relevant, registered) have been explained.

## Data Availability

Data are available on request from authors.
